# Percutaneous Transhepatic Endobiliary Microwave Ablation Before Stenting for Malignant Obstructive Jaundice: Evidence Synthesis and Preliminary Technical Experience

**DOI:** 10.3390/medicina62040611

**Published:** 2026-03-24

**Authors:** Adam Hatzidakis, Nikolas Matthaiou, Leonidas Kougias, Georgios Papadopoulos, Alexandros Mekras, Dimitrios Tsavdaris, Eleni Karlafti, Daniel Paramythiotis

**Affiliations:** 1 Interventional Radiology Unit, Radiology Clinic, AHEPA University Hospital, Aristotle Medical School of Thessaloniki, 54636 Thessaloniki, Greece; 2 Department of General and Visceral Surgery, SHG-Klinikum Merzig, Academic Hospital of University of Saarland, 66663 Merzig, Germany; 3 First Propaedeutic Surgical Clinic, AHEPA University General Hospital, School of Medicine, Aristotle University of Thessaloniki, 54636 Thessaloniki, Greece; 4 First Internal Medicine Department, AHEPA University Hospital, Aristotle Medical School of Thessaloniki, 54636 Thessaloniki, Greece

**Keywords:** microwave ablation, malignant biliary obstruction, cholangiocarcinoma, percutaneous transhepatic biliary intervention, stent patency

## Abstract

Malignant biliary obstruction is commonly treated with biliary stenting either endoscopically or percutaneously; however, tumor ingrowth might occlude the stent, often leading to recurrent jaundice and repeat interventions. Endobiliary microwave ablation (MWA) is an emerging adjunct intended to devitalize intraductal tumors and potentially prolong stent patency. This review assesses the state of the art of endobiliary ablation for malignant biliary obstruction, focusing on the technique and safety of percutaneous procedures, as well as patient outcomes. It also discusses the use of flexible endobiliary MWA for hilar cholangiocarcinoma. The review covers ablation methods such as radiofrequency and MWA, which can be performed endoscopically or percutaneously. Research indicates that endobiliary thermal ablation is technically feasible and can be safely combined with stenting. Some studies suggest it may prolong stent patency and decrease the necessity for repeat procedures compared with stenting alone. Percutaneous techniques may be particularly helpful in complex hilar cases, allowing accurate energy delivery, protection of secondary bile ducts, and tailored stent placement. New microwave systems can heat tissue more deeply and evenly than radiofrequency ablation, which may improve local tumor control. Endobiliary thermal ablation appears to be a useful supplement to stenting, especially for patients with unresectable hilar cholangiocarcinoma. Flexible percutaneous MWA probes could make this treatment more widely available. Still, more high-quality studies are needed to find optimal ablation settings, identify which patients benefit most, and compare this method with standard stenting.

## 1. Introduction

Cholangiocarcinoma is a rare aggressive malignancy of the biliary epithelium, accounting for approximately 10–15% of primary hepatobiliary cancers and 3% of all gastrointestinal malignancies [1]. Risk factors include chronic biliary inflammation (primary sclerosing cholangitis, hepatolithiasis, and choledochal cysts), liver fluke infestation, cirrhosis, hepatitis B and C, and metabolic syndrome, though most cases arise without identifiable risk factors [2,3]. Because of its nonspecific presentation, diagnosis is often delayed, often presenting with biliary obstruction, jaundice, or cholangitis [4]. Natural history is characterized by progressive local invasion, biliary obstruction, and early metastasis, resulting in poor prognosis; median overall survival for unresectable disease is less than one year [5]. Biliary obstruction can however also be caused by pancreatic cancer, the most common being pancreatic ductal adenocarcinoma involving the head of the pancreas, leading to compression of the distal common bile duct [6]. Malignant biliary obstruction (MBO) occurs in the majority of patients with pancreatic cancer and is a frequent initial presentation [7,8]. The natural history is marked by rapid progression, with median survival after diagnosis of MBO typically less than one year, and 6-month mortality rates of 40–60% [8]. Biliary stenting is the mainstay of palliation, but complications such as stent occlusion and cholangitis are common and can further impact chemotherapy completion and survival [7].

Self-expandable metallic stents (SEMSs) are commonly used to relieve jaundice; however, stent failure, recurrent blockages, and the need for additional interventions may occur. To reduce these challenges and prolong stent patency, clinicians are increasingly considering strategies such as covered stents and endobiliary tumor ablation prior to stent placement [9,10]. Endobiliary bipolar radiofrequency ablation (RFA) was among the earliest techniques used in the bile duct to address tumor-related stent failure. RFA destroys tumor tissue at the intended stent site, which may slow tumor progression, particularly when followed by placement of a covered stent. Standard RFA probes treat a limited area, approximately 0.5 cm in diameter. Nevertheless, studies have demonstrated encouraging outcomes. For instance, Uyanik et al. reported a median stent patency of 223 days with RFA, compared to 158 days with stenting alone, supporting the approach of intraductal tumor treatment [11].

More recently, a novel technique using flexible microwave (MW) 14 G probes has been developed for the same indication (MedWaves AveCure) with an active 2.5 cm long tip [12]. This might be more effective by creating a larger necrosis zone of 1.1 × 2.5 cm. Pekçevίk et al. reported a 21-patient study treated by the flexible MW-ablation probe [13]. In both studies, uncovered self-expandable metallic stents were placed post-ablation, without major complications and with good follow-up results [12,13]. In the 21-patient study, a median stent patency of 108 days was reported with three cases of in-stent restenosis [13].

The aim of this review is to summarize and carefully analyze the current evidence on endobiliary thermal ablation as an adjunct to biliary stenting in the management of MBO, with a particular focus on percutaneous techniques. Specifically, this review analyzes the technical aspects, safety profile, and clinical outcomes of endobiliary RFA and MW ablation (MWA), while exploring the emerging role of flexible percutaneous MWA in the treatment of complex hilar cholangiocarcinoma.

## 2. Mechanisms of Stent Failure

In spite of the continued development and usage of SEMS in the palliation of MBO, the issue of unsatisfactory stent patency continues to remain a common problem in clinical practice. The pathophysiological basis of unsatisfactory stent functioning can be varied, including tumor biology, stent design, and biliary anatomy factors [14,15,16].

The ingrowth of the tumor represents one of the most common reasons for the failure of the SEMSs, particularly those that are uncovered. This failure of the uncovered stents to prevent the ingrowth of the tumor results from their porous framework. This framework enables the ingrowth of the tumor cells, thus gradually leading to the obstruction of the stent [14,15,16]. The common occurrence of this mechanism is also one of the reasons for the development of covered stents. Tumor overgrowth is defined as tumor progression beyond the proximal or distal ends of the stent. Overgrowth of the tumor may be associated with the covered and uncovered stents and represents another important factor responsible for the failure of the stents. Overgrowth of the tumor may lead to the obstruction of the stents either due to the compression of the stent on the common duct or due to the obstruction of the upper stent extremity [14,15,16].

Another significant cause of dysfunction of SEMS is sludge and food impaction. The presence of biliary sludge, bacteria, and food debris within the stent lumen can cause partial or complete occlusion of the stent [17,18,19]. This complication is noted with increasing incidence with covered SEMS due to bile flow dynamics and the smooth surface of the covering membrane and is affected by stent size and length. Other reasons for failure of stent and bile duct obstruction are stent migration, primarily with covered SEMSs [14,15,16]; cholecystitis and pancreatitis, caused by interference with cystic and pancreatic duct orifices during stent deployment [16,17]; stent deformity and fracture [20]; and hyperplasia [21].

Covered SEMSs significantly reduce ingrowth but increase the rates of migration and sludge occlusion, while the uncovered stents have a higher ingrowth and epithelial hyperplasia but lower rates of migration. Furthermore, stent length and diameter have a significant effect on the patency of the stent, and longer and smaller stent diameters have higher rates of stent dysfunction and failure [14,22,23].

### Endobiliary Ablation in Malignant Biliary Obstruction

Adjunctive intraductal ablation has emerged as an attempt to locally debulk the tumor, delaying ingrowth and prolonging SEMS function. Most clinical experience to date relates to intraductal RFA. Prospective and retrospective studies have shown that adding intraductal RFA to stenting may improve stent patency, while overall survival benefits appear more variable across cohorts and underlying tumor types [9,10,11,12]. Wang et al. showed that percutaneous intraductal RFA combined with stenting significantly prolonged patency [5.8 (2.8–11.5) months] compared with stenting alone [4.5 (2.4–8.0) months] but did not translate into improved survival [24]. In a larger comparative study, Cui et al. reported longer primary and secondary stent patency with intraluminal RFA plus stenting versus stenting alone, with the magnitude of benefit particularly notable in cholangiocarcinoma subgroups (e.g., primary stent patency 7.4 vs. 4.3 months; secondary stent patency 12.6 vs. 5.0 months) [25]. Early feasibility works also supported acceptable short-term safety profiles, with low rates of major complications and no clear signal of increased procedure-related mortality [10].

More recently, temperature-controlled endobiliary RFA systems have aimed to improve energy delivery consistency and reduce collateral injury; in one comparative study, Uyanık et al. reported improved primary stent patency (223 vs. 158 days) and overall survival (329 vs. 236 days) versus stenting alone, without major complications in the ablation arm, although hemobilia requiring endovascular management occurred in the control arm [11]. Nevertheless, clinically important adverse events, including hemobilia, pseudoaneurysm, and rare bile duct or adjacent bowel perforation, have been reported after endobiliary thermal ablation, emphasizing the need for careful technique, energy selection, and post-procedural surveillance [11,26].

Two researchers (N.M. and D.T.) searched PubMed and Scopus using the keywords “radiofrequency ablation” AND “malignant biliary obstruction” until 25 February 2026. A total of 358 articles were identified, of which six are randomized trials of endobiliary RFA with stenting for MBO [27,28,29,30,31,32]. The results of the randomized trials of endobiliary RFA as an adjunct to stenting for MBO show heterogeneous but clinically relevant outcomes. The beneficial effects of RFA on stent patency are universally found, but they are context-dependent, having a variation from mild improvement to statistically and clinically significant improvement. The improvements are most marked for specific conditions such as extrahepatic cholangiocarcinoma, occluded SEMS, and longer and/or more complex strictures [28,30]. In contrast, pragmatic trials involving scheduled stent exchanges and/or mixed tumor cohorts did not demonstrate a statistically significant stent patency benefit [27,31], suggesting that stent type, tumor and stricture site, and endpoint criteria may all play important roles. The outcomes for overall survival are equally variable. The best evidence for improved survival comes from the larger trials, whereas smaller trials show no clear benefit. Survival benefit does not correlate with improvements in stent patency; thus, it is possible that other important outcomes such as better biliary sepsis control and/or greater ability to tolerate systemic therapies may be relevant. Critically, however, RFA has been shown to be safe and free of adverse effects such as pancreatitis, cholangitis, and perforation across all trials. Although isolated complications such as cholecystitis are reported with RFA [27], these are rare. The evidence to date supports the use of RFA as a technically safe and beneficial intervention for patients with MBO, although the effect clearly depends on the context and specific trials (Table 1).

This search also identified 7 meta-analyses [33,34,35,36,37,38,39] that showed that endobiliary RFA plus stenting shows a relatively consistent association with improved overall survival, whereas the effect on stent patency is less consistent and often absent in time-to-event analyses. Multiple pooled analyses reported an overall survival advantage for RFA + stent compared with stent alone (HR ~0.47–0.65), including analyses that prioritized adjusted hazard ratios and those that combined randomized and observational data. Importantly, even the most methodologically conservative synthesis limited to randomized controlled trials [33] showed improved 6-month survival, supporting the idea that the survival signal is not attributable solely to nonrandomized confounders.

In contrast, pooled stent patency findings depend largely on how patency is measured. Meta-analyses using fixed-time patency rates (e.g., 3- and 6-month patency) often show no overall difference, with subgroup signals emerging in cholangiocarcinoma and portal stenoses at later time points (mostly 6 months). Analyses using time-to-event patency HRs [35,38] generally report no significant difference, suggesting that the patency benefit may be modest, delayed, or limited to specific phenotypes rather than broadly generalizable across malignant etiologies and stent strategies. A key methodological point is that some trials incorporate planned crossovers (particularly with plastic stents), which can compress differences between arms and reduce the ability of meta-analyses to detect a patency advantage. In contrast, in settings where tumor ingrowth/overgrowth is the dominant mechanism of failure (e.g., cholangiocarcinoma and portal disease), pooled subgroup results suggest that intraductal tumor decompression may translate into clinically meaningful subsequent patency differences. Safety results across all meta-analyses are generally reassuring, with no consistent excess risk of pancreatitis, cholangitis, or bleeding. However, a recurring signal across all larger pooled datasets is an increased incidence of cholecystitis in the RFA arm, such as significantly increased odds in Liu et al., 2023 [35], and increased absolute incidence in Ramai et al., 2025 [33]. This pattern of adverse events is clinically plausible (thermal injury or edema near the origin of the cystic duct) and supports careful patient selection (proximity of tumor to the cystic duct), careful energy delivery to the proximal CBD, and early postoperative follow-up. Collectively, the current meta-analyses support the idea that endobiliary RFA is a safe adjunct to stenting with a potential survival benefit, while the patency benefit is variable and appears more apparent in selected subgroups (CCA and/or portal disease, and possibly at later time points). These findings justify continued use in experienced centers and provide a rationale for newer technologies, including MWA, that may offer more homogeneous ablation geometry and potentially stronger effects on intraluminal tumor control and stent function. Cholangioscopy may be useful for preoperative mapping, guiding selective biliary drainage, and facilitating therapeutic interventions such as ablation [40,41], but carries a higher risk of cholangitis than standard ERCP, necessitating prophylactic antibiotics and ensuring adequate drainage [42,43]. Cost and availability may limit its use to specialized centers [43] (Table 2).

Endobiliary MWA is a newer intraductal thermal modality with several theoretical and practical advantages over RFA, including higher intratumoral temperatures, less dependence on tissue impedance, and potentially more homogeneous heating without requiring firm electrode–tissue contact [12,13]. Importantly, these attributes may be relevant in biliary strictures where tissue contact is variable, luminal geometry is constrained, and conductive heat loss to flowing bile or adjacent vascular structures can complicate predictable ablation. Clinical evidence for intraductal MWA remains limited [12]. Pekçevίk et al. and Balli et al. reported MWA combined with SEMS placement for malignant extrahepatic obstruction, again demonstrating feasibility and a signal toward improved patency, while noting that procedure-related complications, including rare, delayed bowel perforation, can occur and may be multifactorial in this fragile population [13]. Differences in reported patency across available MWA studies likely reflect heterogeneity in stricture location (hilar vs. distal), tumor biology, ablation parameters, stent strategies (covered vs. uncovered; unilateral vs. bilateral), and competing risks of death before stent failure [12,13]. The group of patients who may benefit most from a technique like this is patients who have tumors ≥3 cm or tumors adjacent to large vessels, as this group achieves higher intratumoral temperatures and faster ablation times and is less susceptible to the heat-sink effect caused by blood flow in large vessels [44]. Nevertheless, the currently available data are insufficient to draw definitive conclusions regarding technical success, clinical efficacy, or safety profile, and larger prospective studies are required to more clearly define the role of intraductal MWA in MBO.

Several technical considerations deserve emphasis for endobiliary MWA, particularly in hilar strictures. First, device design may limit “over-the-wire” coaxial delivery; our procedure required sheath/wire/catheter strategies to maintain stable access and allow controlled repositioning across the lesion length [13,26]. Second, ablation dosing in the bile duct must balance efficacy against the risk of mural necrosis, vascular injury, and perforation. Preclinical data and early clinical studies suggest that ablation zones can extend beyond the immediate lumen, and ex vivo estimates may not reliably predict in vivo effects because perfusion and heat sink can alter lesion geometry [12]. This uncertainty supports conservative, standardized energy delivery with careful fluoroscopic positioning, avoidance of prolonged overlapping ablations in one focal segment, and routine post-ablation cholangiography to exclude perforation or uncontrolled extravasation. Third, a staged percutaneous strategy (initial external/internal drainage to control cholangitis and improve jaundice, followed by definitive ablation plus stenting) may improve procedural safety by allowing tract maturation and reducing intraductal pressure at the time of thermal therapy.

## 3. Endoscopic Versus Percutaneous Approaches to Endobiliary Ablation

Endobiliary thermal ablation for MBO can be performed using either endoscopic or percutaneous access, with the choice of approach largely determined by tumor location, biliary anatomy, prior interventions, and institutional expertise.

Endoscopic techniques are performed via endoscopic retrograde cholangiopancreatography (ERCP) or endoscopic ultrasound (EUS) and are the preferred option when the anatomy of the biliary tract allows it. These techniques are well-suited for distal biliary strictures, extrahepatic cholangiocarcinoma, and pancreatic head malignancies, with large cohort studies and guideline-based recommendations having demonstrated that endoscopic interventions are associated with lower overall adverse event rates, shorter hospital stays, and reduced healthcare costs compared with percutaneous approaches, especially in pancreatic cancer and in centers with lower percutaneous procedure volumes [45,46]. The American College of Gastroenterology recommends endoscopic biliary drainage and adjunctive therapies as first-line management for MBO whenever technically feasible [47]. However, many limitations commonly prevent the smooth performance of these techniques, such as access to the biliary hilum, particularly in Bismuth type III and IV strictures, and in general in complex hilar anatomy. These cases can be treated with percutaneous techniques [48,49].

Percutaneous approaches, most commonly performed via percutaneous transhepatic cholangiography (PTC), access the biliary system through the liver parenchyma and are typically reserved for patients in whom endoscopic access is not feasible or has failed [46,50,51]. Common indications include surgically altered anatomy, duodenal obstruction, failed ERCP or EUS, and high-grade hilar strictures requiring bilateral or multi-segmental drainage [15,52]. Percutaneous RFA has demonstrated high technical success rates and clinical outcomes comparable to endoscopic approaches in terms of stent patency and survival, but it is also characterized by a higher complication rate [29,33]. Percutaneous access allows direct, stable positioning of ablation probes, facilitates treatment of multiple intrahepatic ducts, and enables tailored bilateral or multi-segmental ablation strategies, which are often required in advanced hilar cholangiocarcinoma [46,50,51].

After two reviewers (N.M. and D.T.) searched PubMed and Scopus using the terms Endoscopic AND Percutaneous AND Biliary Drainage, 5605 results were obtained. Of these, 12 meta-analyses [53,54,55,56,57,58,59,60,61,62,63,64] were included that compared endoscopic and percutaneous approaches for biliary drainage in MBO. Across meta-analyses (Table 3), 30-day mortality is generally similar between endoscopic and percutaneous approaches, while outcomes diverge by anatomy and clinical intent. In mixed MBO populations, technical/therapeutic success is often not significantly different, though sensitivity analyses and “high-level” obstruction subgrouping suggest that PTBD/PTCD may achieve higher drainage success in complex/high-obstruction and hilar disease. Complication patterns are consistent: PTBD/PTCD tends to show lower pancreatitis and/or cholangitis in several pooled analyses, but a higher bleeding/tube-related risk is repeatedly observed in larger datasets. In the specific setting of failed ERCP, pooled comparisons indicate that EUS-guided drainage provides comparable technical success with fewer adverse events and fewer re-interventions than percutaneous drainage, supporting EUS-BD as a preferred rescue option where expertise exists. Finally, in resectable MBO, two independent meta-analyses [57,58] show a robust association between PTBD and higher implantation/seeding metastasis, favoring endoscopic drainage when feasible in potentially curable patients.

## 4. Emerging Percutaneous Microwave Ablation Strategies in Complex Biliary Obstructive Disease: Technical Report

A novel technique for the treatment of complex MBO can be achieved by percutaneous transhepatic intrabiliary MWA with a bipolar probe, along with implantation of a tailored stent that comprises a combination of self-expandable metal stents with and without a covering membrane. The technique combines intrabiliary MWA with metal stent implantation for biliary patency with preservation of ductal outflow.

**Figure 1:**
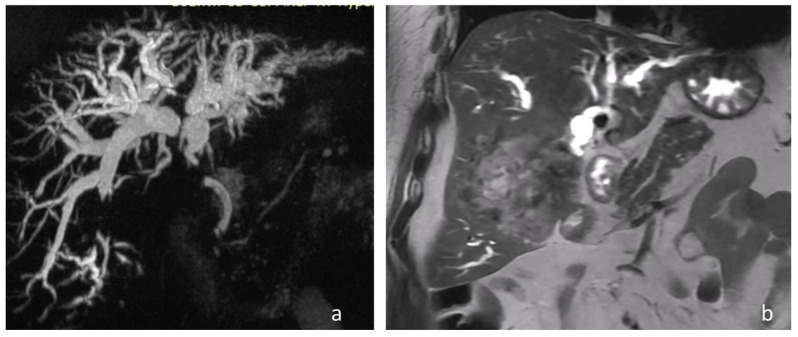
(**a**) MRCP image showing a hilar biliary obstruction with intrahepatic duct dilatation. (**b**) MRI image of a HASTE-Coronal sequence shows a large right liver lobe tumor reaching the liver hilum.

This emerging technique could be a viable method for patients with complex malignant strictures in cases in which endoscopic procedures are either not possible or have already failed. Cross-sectional scans often reveal MBO at the level of the hilum with upstream dilation of the intrahepatic bile ducts, suggesting that there is a rational basis for percutaneous treatment in such patients (Figure 1a,b). By using established percutaneous biliary access techniques, there is potential to perform controlled intraductal ablation of a malignant stricture and then stenting to maintain biliary patency.

**Figure 2:**
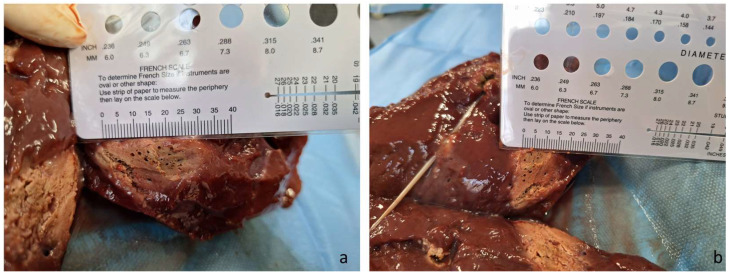
(**a**) Bovine liver after application of microwave ablation with the Amica probe for 5 min at 50 watts. The ablation zone length is about 3 cm. (**b**) Similar image measuring a maximum ablated diameter of about 2 cm.

Recent experience with the newly developed flexible microwave ablation probe (Amica Probe-Flex, HS-Amica, Aprilla, Italy) has further expanded the technical possibilities of this approach. This system allows intrabiliary MWA through standard percutaneous access, followed by covered stent placement with proximal intrahepatic bare-metal stent extension to preserve segmental drainage. In preliminary ex vivo testing, this probe generated an ablation zone measuring approximately 3 cm in length and 2 cm in diameter, supporting its potential to achieve more extensive and homogeneous intraductal tumor devitalization than previously available endobiliary devices (Figure 2a,b).

**Figure 3:**
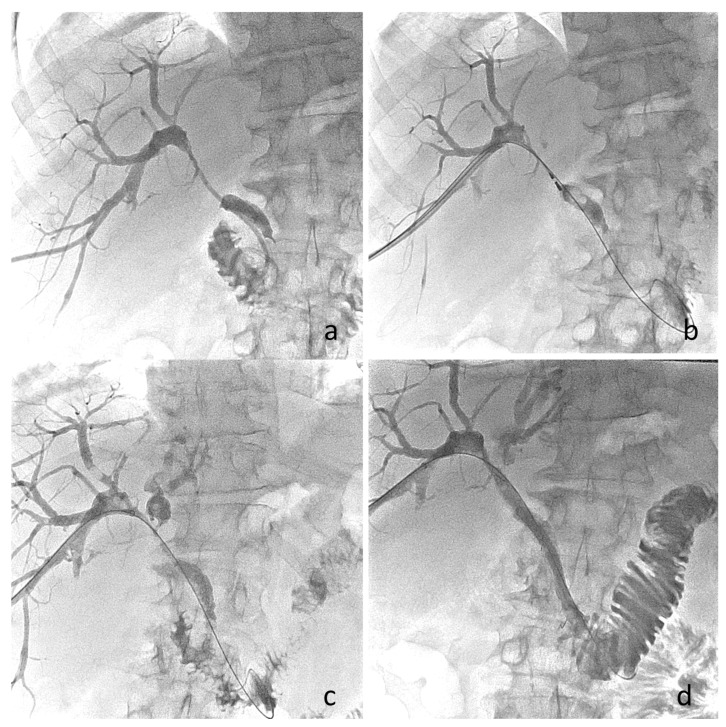
(**a**) Cholangiographic image after placement of an 8 Fr biliary drainage catheter through the 6th liver segment. The CHD is infiltrated at the hilum level, and communication with the left biliary ducts is missing. (**b**) The MW probe is placed at the obstruction zone and one ablation session was applied under conscious sedation. A second “safety” wire was left outside the sheath through which the ablation probe was introduced. (**c**) Post-ablation, cholangiography revealed communication with the left biliary ducts without signs of perforation. (**d**) Cholangiographic image after covered (8.5 Fr) and uncovered (6 Fr) metallic stent placement showing adequate drainage towards the duodenum.

Using the flexible bipolar MWA system compatible with 8 Fr transhepatic sheaths, intraductal ablation can be performed along extended segments of malignant involvement through sequential applications. Each ablation cycle typically treats approximately 2 cm of ductal length, allowing stepwise coverage of longer infiltrative strictures through controlled proximal withdrawal of the probe. The intra-procedure representative images illustrating cholangiography show the position of the probe at the site of obstruction, maintaining access via the guidewire during energy delivery (Figure 3a,b). The intensity parameters used in this procedure have been described as follows: one cycle lasting 5 min at 60 W per segment under conscious sedation.

Immediate post-ablation cholangiography has demonstrated improved duct visibility in previously blocked hilar segments, including restored connections with secondary biliary branches that were not seen before ablation (Figure 3c). These results suggest a quick reopening of the duct or a decrease in the tumor inside the duct at the blockage, which helps with later stent placement.

After ablation, using a hybrid stent approach can be especially helpful in complex hilar anatomy. Placing a covered self-expandable metallic stent across the main tumor area can reduce tumor growth into the stent, while adding an uncovered metallic stent at either end helps keep the segmental and subsegmental bile ducts open. Final cholangiography after both stents are placed shows good bile drainage toward the duodenum and open intrahepatic branches (Figure 3d). This combined method tries to balance the risks: covered stents block tumor growth inside but may close offside branches, while uncovered stents keep side branches open but are more likely to allow tumor ingrowth.

Initial clinical experience with this combined percutaneous intrabiliary MWA and hybrid stenting technique demonstrates technical feasibility, absence of procedure-related complications, and sustained biliary drainage. The primary stent remains patent for durations comparable to or exceeding those reported for endobiliary ablation-assisted stenting. Although current evidence is limited, these findings support further investigation of this technique as a potential adjunct to palliative management of unresectable hilar cholangiocarcinoma.

To the best of current knowledge, the use of percutaneous transhepatic intrabiliary MWA with this flexible bipolar probe, followed by both covered and bare-metal stent placement, has not been previously reported. Further studies are warranted to assess the reproducibility of this method, optimize ablation parameters, and compare its efficacy with existing endobiliary ablation techniques.

From a clinical integration standpoint, endobiliary ablation should be viewed as an adjunct rather than a substitute for durable drainage and systemic therapy. Contemporary reviews and society guidance increasingly recognize that local ablative techniques (RFA and photodynamic therapy) can be incorporated in experienced centers to improve palliation, while underscoring persistent gaps in comparative evidence, optimal timing, and the role of repeat sessions for recurrent obstruction [26]. Prolonged stent patency in MBO enables uninterrupted chemotherapy by reducing recurrent jaundice, cholangitis, and stent-related complications, thereby increasing the likelihood of completing planned treatment and improving survival and quality of life [7,65]. Although prospective studies directly linking stent performance to chemotherapy delivery and quality-of-life outcomes remain needed, for MWA specifically, prospective comparative studies are needed to define optimal power/time settings by stricture location and duct diameter, standardized endpoints (primary/secondary patency, cholangitis-free intervals, and quality-of-life metrics), device-specific safety profiles, and best stent selection strategies (covered, uncovered, and hybrid) in hilar disease. In addition, it will be important to clarify whether MWA offers clinically meaningful advantages over RFA in scenarios where tissue contact is inconsistent (e.g., irregular hilar tumors) or where repeat ablation through an existing SEMS is desired—a technique already applied for RFA recanalization in occluded stents [26,66].

The technical experience presented in this manuscript represents a very preliminary application of percutaneous intrabiliary microwave ablation using a flexible bipolar probe in a limited number of selected cases. At this stage, the primary aim is to demonstrate technical feasibility and procedural workflow rather than to provide definitive conclusions regarding efficacy. Given the small sample size and relatively short follow-up, reliable estimates of stent patency duration, long-term survival, and complication rates cannot yet be established. Accordingly, this approach should be considered exploratory. Larger prospective studies with standardized ablation parameters and systematic follow-up are necessary to better define clinical outcomes, safety profile, and durability of this technique.

The available evidence on intrabiliary MWA is limited by small sample sizes, predominantly retrospective designs, and significant heterogeneity in tumor types, stricture characteristics, and stent configurations. Many published MWA studies include fewer than 30 patients and are subject to selection bias, while survival analyses are confounded by competing mortality in advanced malignancy, making it difficult to determine the true clinical impact of improved biliary patency. Additionally, outcomes vary between cholangiocarcinoma, pancreatic cancer, and other malignancies, yet these entities are often analyzed together. Differences in stent type (plastic versus SEMS and covered versus uncovered) and unilateral versus bilateral deployment further complicate comparisons. Most importantly, no randomized comparative trials evaluating MWA exist, and the current level of evidence remains low; therefore, the potential survival benefit and long-term efficacy of this approach remain uncertain and require prospective validation.

## 5. Conclusions

Endobiliary thermal ablation, particularly when combined with covered stent placement, represents a promising adjunctive strategy for the palliative management of malignant biliary obstruction. Percutaneous microwave ablation offers technical feasibility and potential advantages in obstructive biliary disease, but current evidence remains limited, underscoring the need for prospective, comparative studies to define its optimal role alongside standard stenting.

## Figures and Tables

**Table 1:** Randomized controlled trials evaluating endobiliary RFA combined with stenting versus stenting alone for malignant biliary obstruction.

Study	Population	N (RFA vs. Control)	Stent Type	Primary Patency	Overall Survival	Major Adverse Events
Gao et al., 2021 [27]	Locally advanced or metastatic cholangiocarcinoma and ampullary carcinoma	87 vs. 87	Plastic stents	4.1 vs. 3.7 months; *p* = 0.674	14.3 vs. 9.2 months; HR ≈ 0.49; *p* < 0.001	Higher acute cholecystitis in RFA group (10.3% vs. 0%); otherwise, similar
Yang et al., 2018 [28]	Unresectable extrahepatic cholangiocarcinoma (non-Bismuth III–IV)	32 vs. 33	SEMS	6.8 vs. 3.4 months; *p* = 0.02	13.2 vs. 8.3 months; *p* < 0.001	6.3% [2/32] vs. 9.1% [3/33]; *p* = 0.67
Kang et al., 2021 [29]	Unresectable extrahepatic cholangiocarcinoma	24 vs. 24	SEMS	132 vs. 116 days; *p* = 0.440	244 vs. 180 days; *p* = 0.281	4.2% vs. 12.5%, *p* = 0.609
Kang et al., 2022 [30]	Malignant hilar obstruction	15 vs. 15	SEMS	178 vs. 122 days; *p* = 0.154; benefit in long strictures ≥ 11 mm (*p* = 0.028)	230 vs. 144 days; *p* = 0.643	No excess major complications
Andrasina et al., 2021 [31]	Histologically verified malignant stenosis of bile ducts	36 vs. 40	SEMS	5.2 vs. 4.8 months; *p* = 0.79	6.8 vs. 5.2 months; *p* = 0.495	No excess major complications
Albers et al., 2022 [32]	Unresectable distal malignant biliary obstruction	44 vs. 42	SEMS	3 months: 81.8% vs. 73.1%; *p* = 1.0 6 months: 52.4% vs. 33.3%; *p* = 0.6	HR = 0.72; *p* = 0.389 for RFA + SEMS	2.3% vs. 10.5%; *p* = 0.18

Abbreviations: RFA, radiofrequency ablation; SEMS, self-expandable metal stent.

**Table 2:** Meta-analyses evaluating endobiliary radiofrequency ablation (RFA) plus stenting versus stenting alone for malignant biliary obstruction (MBO).

Study	Included Studies/Patients	Key Comparisons	Overall Survival (OS)	Stent Patency	Adverse Events (AEs)
Ramai et al., 2025 [33]	9 RCTs; 750 pts (374 RFA+stent vs. 376 stent)	RFA+stent vs. stent	Improved 6-month survival (RR 0.84, 95%CI 0.73–0.96; I^2^ = 21%; *p* = 0.01); OS benefit noted in cholangiocarcinoma subgroup	No difference in 3-month patency (RR 1.01, 95%CI 0.92–1.11; I^2^ = 4%); no effect by plastic vs. uncovered metal subgroup	Higher cholecystitis (5.1% vs. 0.3%); otherwise not highlighted as increased
Veras et al., 2024 [34]	6 studies; 439 pts	RFA+stent vs. stent	Improved OS (pooled MD +85.70 days, 95%CI 34.29–137.10; I^2^ = 98%; *p* = 0.001). Survival rate: no difference at 3 mo; better at 6 mo (RD 0.17, 95%CI 0.09–0.25; I^2^ = 0%; *p* < 0.001). CCA subgroup OS benefit (MD +83.14 days)	Overall patency time: NS (MD +22.25 days; I^2^ = 97%). Patency rates: NS at 3 mo (RD 0.04; *p* = 0.56) and 6 mo (RD 0.03; *p* = 0.66) overall; CCA subgroup showed longer patency (MD +79.25 days) and improved 6 mo patency rate (RD 0.13; *p* = 0.02); hilar subgroup improved patency time (MD +83.71 days) and 6 mo patency rate (RD 0.15; *p* = 0.04)	Total ERCP-related AEs: no difference (RD 0.03; I^2^ = 26%; *p* = 0.44)
Liu et al., 2023 [35]	11 studies; 1283 pts (434 RFA+stent vs. 849 stent)	RFA+stent vs. stent	Improved OS (pooled HR 0.65, 95%CI 0.58–0.73; I^2^ = 40%; *p* < 0.00001)	Patency duration: no significant difference (pooled HR 1.04, 95%CI 0.84–1.28; I^2^ = 46%; 6 studies)	Pancreatitis, cholangitis, hemorrhage: no difference; cholecystitis increased (OR 11.34, 95%CI 2.88–44.59; *p* = 0.0005). All AEs modestly higher (OR 1.41, 95%CI 1.02–1.96)
Song et al., 2022 [36]	33 studies; 2974 pts	PDT+stent vs. RFA+stent vs. stent	Both PDT+stent and RFA+stent improved OS vs. stent alone; ranking: PDT+stent most likely best for OS	RFA+stent improved mean patency vs. stent alone (MD ~2.0; 95%CI 1.1–2.8); ranking: RFA+stent most likely best for patency	Mild bleeding/cholangitis/pancreatitis similar across modalities
Song et al., 2022 [37]	19 studies; 1946 pts (764 RFA+stent vs. 1182 stent); includes 3 RCTs + 16 non-RCTs	RFA+stent vs. stent	Improved OS (HR 0.55, 95%CI 0.48–0.63; I^2^ = 2%). RCT subgroup OS benefit maintained (HR 0.41)	Mean patency time longer overall; RCT subgroup: no significant difference for mean patency or patency rates at 3/6 mo	No significant differences in abdominal pain, mild bleeding, cholangitis, pancreatitis
Cha et al., 2021 [38]	8 studies; 420 pts (190 RFA+stent vs. 230 stent); includes 3 RCTs + retrospective/percutaneous mix	RFA+stent vs. stent	Improved OS (pooled HR 0.47, 95%CI 0.34–0.64; I^2^ = 44%)	Patency: NS (pooled HR 0.79, 95%CI 0.57–1.09; I^2^ = 7%; only 4 studies)	Mild-to-moderate AEs; no significant between-group AE differences reported
Zeng et al., 2016 [39]	9 studies; 263 pts	Feasibility/outcomes after RFA	Pooled mortality (30d ~1.5%, 90d ~20.9%, 2y ~48.1%); pooling OS by HR not feasible due to reporting heterogeneity	Median patency pooled descriptively (~7.6 months)	Pooled AE rate ~17% (95%CI 10–25%); notable rare severe AEs described (e.g., delayed bleeding and liver infarction in advanced hilar cases)

Abbreviations: AE, adverse event; CCA, cholangiocarcinoma; CI, confidence interval; ERCP, endoscopic retrograde cholangiopancreatography; I^2^, heterogeneity statistic; MD, mean difference; MBO, malignant biliary obstruction; OS, overall survival; PDT, photodynamic therapy; pts, patients; RCT, randomized controlled trial; RD, risk difference; RR, risk ratio; SEMS, self-expandable metal stent; NS, not significant. (1) Meta-analyses differ in inclusion criteria (RCT-only vs. mixed observational designs), endpoints (time-to-event HR vs. fixed-time patency/survival rates), and stent strategies (plastic vs. SEMS), which should be considered when interpreting pooled estimates. (2) Direction of effect: HR < 1 favors RFA+stent for OS; patency HR interpretation varies across publications (some report HR for stent dysfunction rather than patency). Where the original authors reported “no significant difference”, results are summarized as NS.

**Table 3:** Meta-analyses comparing endoscopic (EBD/ERCP/EUS-BD) versus Percutaneous transhepatic drainage (PTBD/PTCD/PTC) in malignant biliary obstruction (MBO).

Study	Comparison	Included Studies/Patients	Technical/Clinical Success	Complications/Re-Intervention	Mortality	Oncologic Outcomes (Seeding/Implantation)
Leng et al., 2014 [53]	PTBD vs. EBD	3 studies; 183 pts	Success NS overall (OR 2.34; NS); sensitivity favored PTBD (OR 5.48)	Overall complications NS	30-day mortality NS (OR 1.29)	—
Zhao et al., 2015 [54]	PTBD vs. EBD	8 studies; 692 pts	Success NS overall (OR 2.18; NS); after excluding outliers, PTBD favored (OR 4.45)	Cholangitis lower with PTBD (OR 0.55); pancreatitis NS; overall complications NS	30-day mortality NS (OR 1.32)	—
Moole et al., 2016 [55]	PTBD vs. EBD	9 studies; 546 pts	Higher successful drainage with PTBD (OR 2.53); advanced hilar CCA subgroup OR 4.94	Cholangitis lower with PTBD; post-papillotomy bleeding higher with PTBD	30-day mortality NS	—
Duan et al., 2017 [56]	PTBD vs. EBD	14 studies; 10,346 pts	Pooled success favored PTBD, but RCT-only: no difference	PTBD lower cholangitis/pancreatitis; higher bleeding/tube dislocation	30-day mortality NS (overall and RCT-only)	—
Wang et al., 2019 [57]	PTBD vs. EBD	10 studies; 2464 pts	—	—	—	Seeding metastasis lower with EBD (10.5% vs. 22.0%; OR 0.35)
Yang et al., 2020 [58]	PTBD vs. EBD	10 studies; 2464 pts	Surgical success NS (OR 1.52; NS)	—	—	Implantation metastasis lower with EBD (11.2% vs. 21.2%; OR 0.35); both catheter-related and peritoneal lower
Rizzo et al., 2020 [59]	PTBD vs. EBD	17 studies; PTBD 2353 vs. EBD 8178	Technical success NS (OR 2.15; NS; high heterogeneity)	PTBD lower pancreatitis (OR 0.14) and cholangitis (OR 0.52); bleeding higher (OR 1.78); tube dislocation NS	30-day mortality NS (OR 1.33)	—
Wang et al., 2024 [60]	PTCD vs. ERCP	21 studies; 1693 pts	Low level: success NS; high level: PTCD higher success (OR 5.27); overall success favored PTCD (OR 2.05)	Overall complications NS (OR 1.64; NS). Jaundice remission: ERCP better in low level, PTCD better in high level; efficacy: ERCP better in low level, PTCD better in high level	NS	—
Hayat et al., 2022 [61]	EUS-BD vs. PTC	10 studies; 1131 pts	Technical success NS; clinical success NS	Fewer acute + total AEs with EUS-BD; lower re-intervention with EUS-BD	Death rate NS (≈1.4% both)	—
Wang et al., 2022 [62]	EUS-BD vs. PTCD	9 studies; 469 pts	Technical success NS; clinical success higher with EUS-BD (overall)	AEs lower with EUS-BD (OR 0.33)	NS	—
Qiu et al., 2025 [63]	PTBD vs. EBD	5 studies; 721 samples	Technical success NS	Differences in post-drainage complications and pancreatitis reported as significant in their analysis; bleeding NS	NS	Implant transfer rate NS
Sun et al., 2025 [64]	Multiple PBD strategies (incl. PTBD)	81 trials; 26,251 pts	—	PBD increased total adverse events overall; cholangitis signal in standard meta-analysis; network suggests ranking differences across drainage modalities	Short-term mortality overall NS	—

Abbreviations: AEs, adverse events; CCA, cholangiocarcinoma; EBD, endoscopic biliary drainage; ERCP, endoscopic retrograde cholangiopancreatography; EUS-BD, endoscopic ultrasound-guided biliary drainage; MBO, malignant biliary obstruction; NS, not significant; OR, odds ratio; PBD, preoperative biliary drainage; PTBD/PTCD/PTC, percutaneous transhepatic biliary drainage/cholangiography/drainage; RCT, randomized controlled trial. (1) Meta-analyses pool heterogeneous populations (distal vs. hilar; pancreatic vs. biliary primaries; palliative vs. preoperative intent), which explains substantial between-study heterogeneity and occasional publication bias. (2) When reported, OR > 1 favors PTBD/PTCD for success outcomes; OR < 1 favors PTBD/PTCD for complication outcomes (lower odds). (3) Seeding/implantation metastasis outcomes apply to resected/potentially curable cohorts and should be interpreted separately from purely palliative drainage studies.

## Data Availability

The original contributions presented in this study are included in the article. Further inquiries can be directed to the corresponding author.

## Abbreviations

The following abbreviations are used in this manuscript:

RF	Radiofrequency
MW	Microwave
CHD	Common Hepatic Duct
MBO	Malignant Biliary Obstruction
SEMSs	Self-Expanding Metal Stents
RFA	Radiofrequency Ablation
MWA	Microwave Ablation

## References

[1] Bergquist A., von Seth E. (2015). Epidemiology of cholangiocarcinoma. Best Pract. Res. Clin. Gastroenterol..

[2] Razumilava N., Gores G.J. (2014). Cholangiocarcinoma. Lancet.

[3] Khan S.A., Toledano M.B., Taylor-Robinson S.D. (2008). Epidemiology, risk factors, and pathogenesis of cholangiocarcinoma. HPB.

[4] Valle J.W., Kelley R.K., Nervi B., Oh D.Y., Zhu A.X. (2021). Biliary tract cancer. Lancet.

[5] Izquierdo-Sanchez L., Lamarca A., La Casta A., Buettner S., Utpatel K., Klümpen H.J., Adeva J., Vogel A., Lleo A., Fabris L. (2022). Cholangiocarcinoma landscape in Europe: Diagnostic, prognostic and therapeutic insights from the ENSCCA Registry. J. Hepatol..

[6] Boulay B.R., Parepally M. (2014). Managing malignant biliary obstruction in pancreas cancer: Choosing the appropriate strategy. World J. Gastroenterol..

[7] Gasparini G., Aleotti F., Palucci M., Belfiori G., Tamburrino D., Partelli S., Orsi G., Macchini M., Archibugi L., Capurso G. (2023). The role of biliary events in treatment and survival of patients with advanced pancreatic ductal adenocarcinoma. Dig. Liver Dis..

[8] Chan A.S. (2022). Gastrointestinal cancer—Gastroesophageal, pancreatic, and hepatobiliary. J. Clin. Oncol..

[9] Krokidis M., Fanelli F., Orgera G., Bezzi M., Passariello R., Hatzidakis A. (2010). Percutaneous treatment of malignant jaundice due to extrahepatic cholangiocarcinoma: Covered Viabil stent versus uncovered Wallstents. Cardiovasc. Intervent. Radiol..

[10] Mizandari M., Pai M., Xi F., Valek V., Tomas A., Quaretti P., Golfieri R., Mosconi C., Guokun A., Kyriakides C. (2013). Percutaneous intraductal radiofrequency ablation is a safe treatment for malignant biliary obstruction: Feasibility and early results. Cardiovasc. Intervent. Radiol..

[11] Uyanık S.A., Öğüşlü U., Atlı E., Yılmaz B., Çevik H., Gümüş B. (2021). Percutaneous endobiliary ablation of malignant biliary strictures with a novel temperature-controlled radiofrequency ablation device. Diagn. Interv. Radiol..

[12] Uyanık S.A., Öğüşlü U., Yılmaz B., Çevik H., Atlı E., Gümüş B. (2020). Percutaneous intraductal microwave ablation of malignant biliary strictures: Initial experience. AJR Am. J. Roentgenol..

[13] Pekçevίk R., Ballı Ö. (2021). Percutaneous intraductal microwave ablation and self-expandable metallic stenting: A new treatment method for malignant extrahepatic biliary obstruction. Cardiovasc. Intervent. Radiol..

[14] Li J., Li T., Sun P., Yu Q., Wang K., Chang W., Song Z., Zheng Q. (2016). Covered versus Uncovered Self-Expandable Metal Stents for Managing Malignant Distal Biliary Obstruction: A Meta-Analysis. PLoS ONE.

[15] Fairchild A.H., Hohenwalter E.J., Gipson M.G., Al-Refaie W.B., Braun A.R., Cash B.D., Kim C.Y., Pinchot J.W., Scheidt M.J., Expert Panel on Interventional Radiology (2019). ACR Appropriateness Criteria® Radiologic Management of Biliary Obstruction. J. Am. Coll. Radiol..

[16] Tsuyuguchi T., Takada T., Miyazaki M., Miyakawa S., Tsukada K., Nagino M., Kondo S., Furuse J., Saito H., Suyama M. (2008). Stenting and interventional radiology for obstructive jaundice in patients with unresectable biliary tract carcinomas. J. Hepatobiliary Pancreat. Surg..

[17] Tamura T., Yamai T., Uza N., Yamasaki T., Masuda A., Tomooka F., Maruyama H., Shigekawa M., Ogura T., Kuriyama K. (2024). Adverse events of self-expandable metal stent placement for malignant distal biliary obstruction: A large multicenter study. Gastrointest. Endosc..

[18] Suksai N., Kamalaporn P., Chirnaksorn S., Siriyotha S. (2023). Factors associated with patency of self-expandable metal stents in malignant biliary obstruction. BMC Gastroenterol..

[19] Mukai T., Iwata K., Iwashita T., Doi S., Kawakami H., Okuno M., Maruta A., Uemura S., Shimizu M., Yasuda I. (2024). Comparison of covered self-expandable metallic stents with 12-mm and 10-mm diameters for unresectable malignant distal biliary obstructions: A prospective randomized trial. Gastrointest. Endosc..

[20] Kim S.H., Oh C.H., Lee J.M., Choi S.J., Choi H.S., Kim E.S., Keum B., Jeen Y.T., Chun H.J., Lee H.S. (2020). Early malfunction of a biliary self-expandable metal stent with an antireflux valve: A case report. Medicine.

[21] Baron T.H. (2001). Expandable metal stents for the treatment of cancerous obstruction of the gastrointestinal tract. N. Engl. J. Med..

[22] Conio M., Mangiavillano B., Caruso A., Filiberti R.A., Baron T.H., De Luca L., Signorelli S., Crespi M., Marini M., Ravelli P. (2018). Covered versus uncovered self-expandable metal stent for palliation of primary malignant extrahepatic biliary strictures: A randomized multicenter study. Gastrointest. Endosc..

[23] Tringali A., Hassan C., Rota M., Rossi M., Mutignani M., Aabakken L. (2018). Covered vs. uncovered self-expandable metal stents for malignant distal biliary strictures: A systematic review and meta-analysis. Endoscopy.

[24] Wang J., Zhao L., Zhou C., Gao K., Huang Q., Wei B., Gao J. (2016). Percutaneous intraductal radiofrequency ablation combined with biliary stent placement for nonresectable malignant biliary obstruction improves stent patency but not survival. Medicine.

[25] Cui W., Wang Y., Fan W., Lu M., Zhang Y., Yao W., Li J. (2017). Comparison of intraluminal radiofrequency ablation and stents vs. stents alone in the management of malignant biliary obstruction. Int. J. Hyperth..

[26] Kim M., Parekh D., Kahaleh M. (2024). Ablation Therapy of the Biliary Tree: Status and Comprehensive Review. J. Clin. Gastroenterol..

[27] Gao D.J., Yang J.F., Ma S.R., Wu J., Wang T.T., Jin H.B., Xia M.X., Zhang Y.C., Shen H.Z., Ye X. (2021). Endoscopic radiofrequency ablation plus plastic stent placement versus stent placement alone for unresectable extrahepatic biliary cancer: A multicenter randomized controlled trial. Gastrointest. Endosc..

[28] Yang J., Wang J., Zhou H., Zhou Y., Wang Y., Jin H., Lou Q., Zhang X. (2018). Efficacy and safety of endoscopic radiofrequency ablation for unresectable extrahepatic cholangiocarcinoma: A randomized trial. Endoscopy.

[29] Kang H., Chung M.J., Cho I.R., Jo J.H., Lee H.S., Park J.Y., Park S.W., Song S.Y., Bang S. (2021). Efficacy and safety of palliative endobiliary radiofrequency ablation using a novel temperature-controlled catheter for malignant biliary stricture: A single-center prospective randomized phase II TRIAL. Surg. Endosc..

[30] Kang H., Han S.Y., Cho J.H., Kim E.J., Kim D.U., Yang J.K., Jeon S., Park G., Lee T.H. (2022). Efficacy and safety of temperature-controlled intraductal radiofrequency ablation in advanced malignant hilar biliary obstruction: A pilot multicenter randomized comparative trial. J. Hepatobiliary Pancreat. Sci..

[31] Andrasina T., Rohan T., Panek J., Kovalcikova P., Kunovsky L., Ostrizkova L., Valek V. (2021). The combination of endoluminal radiofrequency ablation and metal stent implantation for the treatment of malignant biliary stenosis—Randomized study. Eur. J. Radiol..

[32] Albers D., Schmidt A., Schiemer M., Caca K., Wannhoff A., Sauer P., Wiesweg M., Schumacher B., Dechene A. (2022). Impact of endobiliary radiofrequency ablation on biliary drainage in patients with malignant biliary strictures treated with uncovered self-expandable metal stents: A randomized controlled multicenter trial. Gastrointest. Endosc..

[33] Ramai D., Maida M., Smith E.R., Wang Y., Spadaccini M., Previtera M., Chandan S., Huang Y., Tokmak S., Bhandari P. (2025). Endoluminal radiofrequency ablation with stenting versus stenting alone in patients with malignant biliary obstruction: A meta-analysis of randomized trials. Endoscopy.

[34] de Oliveira Veras M., de Moura D.T.H., McCarty T.R., de Oliveira G.H.P., Gomes R.S.A., Landim D.L., Nunes F.G., Franzini T.A.P., Lera Dos Santos M.E., Bernardo W.M. (2024). Intraductal radiofrequency ablation plus biliary stent versus stent alone for malignant biliary obstruction: A systematic review and meta-analysis. Endosc. Int. Open.

[35] Liu C., Dong J., Liu Y., Zhang S., Chen R., Tang H. (2023). Is endoscopic radiofrequency ablation plus stent placement superior to stent placement alone for the treatment of malignant biliary obstruction? A systematic review and meta-analysis. J. Int. Med. Res..

[36] Song S., Gong S., Lei T., Tian H., Lu T., Lei C., Jin H., Yang W., Yang K., Guo T. (2022). Comparative efficacy and safety of local palliative therapeutics for unresectable malignant biliary obstruction: A Bayesian network meta-analysis. Expert Rev. Gastroenterol. Hepatol..

[37] Song S., Jin H., Cheng Q., Gong S., Lv K., Lei T., Tian H., Li X., Lei C., Yang W. (2022). Local palliative therapies for unresectable malignant biliary obstruction: Radiofrequency ablation combined with stent or biliary stent alone? An updated meta-analysis of nineteen trials. Surg. Endosc..

[38] Cha B.H., Jang M.J., Lee S.H. (2021). Survival Benefit of Intraductal Radiofrequency Ablation for Malignant Biliary Obstruction: A Systematic Review with Meta-Analysis. Clin. Endosc..

[39] Zheng X., Bo Z.Y., Wan W., Wu Y.C., Wang T.T., Wu J., Gao D.J., Hu B. (2016). Endoscopic radiofrequency ablation may be preferable in the management of malignant biliary obstruction: A systematic review and meta-analysis. J. Dig. Dis..

[40] Navaneethan U., Moon J.H., Itoi T. (2019). Biliary interventions using single-operator cholangioscopy. Dig. Endosc..

[41] Pereira S.P., Goodchild G., Webster G.J.M. (2018). The endoscopist and malignant and non-malignant biliary obstruction. Biochim. Biophys. Acta Mol. Basis Dis..

[42] Angsuwatcharakon P., Kulpatcharapong S., Moon J.H., Ramchandani M., Lau J., Isayama H., Seo D.W., Maydeo A., Wang H.P., Nakai Y. (2022). Consensus guidelines on the role of cholangioscopy to diagnose indeterminate biliary stricture. HPB.

[43] Fujii-Lau L.L., Thosani N.C., Al-Haddad M., Acoba J., Wray C.J., Zvavanjanja R., Amateau S.K., Buxbaum J.L., Wani S., Calderwood A.H. (2023). American Society for Gastrointestinal Endoscopy guideline on role of endoscopy in the diagnosis of malignancy in biliary strictures of undetermined etiology: Methodology and review of evidence. Gastrointest. Endosc..

[44] Izzo F., Granata V., Grassi R., Fusco R., Palaia R., Delrio P., Carrafiello G., Azoulay D., Petrillo A., Curley S.A. (2019). Radiofrequency Ablation and Microwave Ablation in Liver Tumors: An Update. Oncologist.

[45] Tavakkoli A., Elmunzer B.J., Waljee A.K., Murphy C.C., Pruitt S.L., Zhu H., Rong R., Kwon R.S., Scheiman J.M., Rubenstein J.H. (2021). Survival analysis among unresectable pancreatic adenocarcinoma patients undergoing endoscopic or percutaneous interventions. Gastrointest. Endosc..

[46] Inamdar S., Slattery E., Bhalla R., Sejpal D.V., Trindade A.J. (2016). Comparison of Adverse Events for Endoscopic vs Percutaneous Biliary Drainage in the Treatment of Malignant Biliary Tract Obstruction in an Inpatient National Cohort. JAMA Oncol..

[47] Elmunzer B.J., Maranki J.L., Gómez V., Tavakkoli A., Sauer B.G., Limketkai B.N., Brennan E.A., Attridge E.M., Brigham T.J., Wang A.Y. (2023). ACG Clinical Guideline: Diagnosis and Management of Biliary Strictures. Am. J. Gastroenterol..

[48] Chaudhary U., Shah S.L. (2025). Advances in Endoscopic Diagnosis and Management of Cholangiocarcinoma. J. Clin. Med..

[49] Natha C., Vemulapalli V., Thosani N. (2025). Endoscopic Ablation in Cholangiocarcinoma. Cancers.

[50] Lubbe J., Lindemann J., Gondo W., Kolev N., Aclavio P., Hofmeyr S., Jonas E. (2022). Endoscopic versus percutaneous intervention for palliation in malignant hilar bile duct obstruction—A comparative cohort study. HPB.

[51] Kong Y.L., Zhang H.Y., Liu C.L., He X.J., Zhao G., Wang C., Kong L.H., Zhao J. (2022). Improving biliary stent patency for malignant obstructive jaundice using endobiliary radiofrequency ablation: Experience in 150 patients. Surg. Endosc..

[52] Mizandari M., Kumar J., Pai M., Chikovani T., Azrumelashvili T., Reccia I., Habib N. (2018). Interventional radiofrequency ablation: A promising therapeutic modality in the management of malignant biliary and pancreatic duct obstruction. J. Cancer.

[53] Leng J.J., Zhang N., Dong J.H. (2014). Percutaneous transhepatic and endoscopic biliary drainage for malignant biliary tract obstruction: A meta-analysis. World J. Surg. Oncol..

[54] Zhao X.Q., Dong J.H., Jiang K., Huang X.Q., Zhang W.Z. (2015). Comparison of percutaneous transhepatic biliary drainage and endoscopic biliary drainage in the management of malignant biliary tract obstruction: A meta-analysis. Dig. Endosc..

[55] Moole H., Dharmapuri S., Duvvuri A., Dharmapuri S., Boddireddy R., Moole V., Yedama P., Bondalapati N., Uppu A., Yerasi C. (2016). Endoscopic versus Percutaneous Biliary Drainage in Palliation of Advanced Malignant Hilar Obstruction: A Meta-Analysis and Systematic Review. Can. J. Gastroenterol. Hepatol..

[56] Duan F., Cui L., Bai Y., Li X., Yan J., Liu X. (2017). Comparison of efficacy and complications of endoscopic and percutaneous biliary drainage in malignant obstructive jaundice: A systematic review and meta-analysis. Cancer Imaging.

[57] Wang L., Lin N., Xin F., Ke Q., Zeng Y., Liu J. (2019). A systematic review of the comparison of the incidence of seeding metastasis between endoscopic biliary drainage and percutaneous transhepatic biliary drainage for resectable malignant biliary obstruction. World J. Surg. Oncol..

[58] Yang G., Xiong Y., Sun J., Tang T., Li W., Wang G., Li J. (2020). Effects of different preoperative biliary drainage methods for resected malignant obstruction jaundice on the incidence rate of implantation metastasis: A meta-analysis. Oncol. Lett..

[59] Rizzo A., Ricci A.D., Frega G., Palloni A., de Lorenzo S., Abbati F., Mollica V., Tavolari S., di Marco M., Brandi G. (2020). How to Choose Between Percutaneous Transhepatic and Endoscopic Biliary Drainage in Malignant Obstructive Jaundice: An Updated Systematic Review and Meta-analysis. In Vivo.

[60] Wang Y., Zhao X., She Y., Kang Q., Chen X. (2024). The clinical efficacy and safety of different biliary drainage in malignant obstructive jaundice: A meta-analysis. Front. Oncol..

[61] Hayat U., Bakker C., Dirweesh A., Khan M.Y., Adler D.G., Okut H., Leul N., Bilal M., Siddiqui A.A. (2022). EUS-guided versus percutaneous transhepatic cholangiography biliary drainage for obstructed distal malignant biliary strictures in patients who have failed endoscopic retrograde cholangiopancreatography: A systematic review and meta-analysis. Endosc. Ultrasound.

[62] Wang Y., Lyu Y., Li T., Wang B., Cheng Y. (2022). Comparing Outcomes Following Endoscopic Ultrasound-Guided Biliary Drainage Versus Percutaneous Transhepatic Biliary Drainage for Malignant Biliary Obstruction: A Systematic Review and Meta-Analysis. J. Laparoendosc. Adv. Surg. Tech. A.

[63] Qiu F., Yang T., Han W. (2025). Comparison of Biliary Drainage Techniques for MBO: A Meta-Analysis. Pancreas.

[64] Sun P., Zhong Y., Hu Y., Diwas S., Wu J., Zou R., Zhai A., Yang S., Shi X., Jin Y. (2025). Preoperative biliary drainage for patients with malignant obstructive jaundice: An update on the systematic review and model-based Bayesian network meta-analysis. Int. J. Surg..

[65] Kim J., Gwon D.I., Kim J., Ko E., Kim J.H., Ko G.Y., Yoon H.K. (2026). Percutaneous metallic stent placement for malignant extrahepatic biliary obstruction: Single-center experience in 612 patients. Acta Radiol..

[66] Tomas R., Andrašina T., Matkulcik P., Bernard V., Valek V. (2022). Percutaneous Endoluminal Radiofrequency Ablation of Occluded Biliary Metal Stent in Malignancy Using Monopolar Technique: A Feasibility Study. Cardiovasc. Intervent. Radiol..

